# A Case of Locomotor Ataxy

**Published:** 1866-09

**Authors:** Henry T. Godfrey

**Affiliations:** Benton, Wisconsin


*A Case of Locomotor Ataxy.* By HENRY T. GODFREY, M. D., of Benton, Wisconsin.

P. McD——, aged 32, commercial traveler, of rather intemperate habits, applied to me about the 1st March of this year for medical treatment. His history is as follows: About four years ago, while traveling in Ireland, was attacked by paralysis after a severe horseback ride. Had felt stiff and uncomfortable for several weeks previous, but attributed it to fatigue. Was treated by an eminent Dublin physician with cold baths and tinct. nucis vom., and had so far recovered at the end of two months as to be able to walk about and resume some of the lighter branches of his business, but he has never since entirely recovered the power of his lower extremities. When first I saw him he complained of a tingling sensation from the knees downward; loss to a considerable extent of the co-ordinating power in walking, which obliged him to walk with a cane; inability to walk at night or with his eyes shut; his legs, as he expresses it himself, "fly in all directions." These symptoms are much aggravated by excitement or the use of alcoholic stimulants. His general health is good, but he complains that his memory is not quite so good as it was sometime ago, and thinks it is getting worse.

I advised the use of the galvanic battery, and prescribed tinct. ferri sesqui-chlor. gtt. xx., strychniæ, gr. $\frac{1}{24}$ three times a day. After a few days this mixture began to disagree with his stomach, producing nausea, loss of appetite, etc. I then discontinued the tinct. ferri, dissolving the strychnia in tinct. card. co. by the aid of a few drops of dilute nitric acid. The dose of strychnia was gradually increased to $\frac{1}{12}$ of a grain three times a day, and continued till it produced spasmodic twitchings of the muscles. The battery used was Hall's galvano-magnetic. The current was passed from the last dorsal vertebra alternately to each foot twice a day. Cold sponge bath used every morning and total abstinence from excitement and intoxicating liquor, with a liberal diet, was enjoined.

He continued this treatment for about three months without any very marked improvement after the first two weeks. At the end of the third month of treatment, he won a lawsuit, a success which proved too much for his temperate habits, so he went on a systematic spree for about two weeks,—coming to town in the morning and hiring a conveyance to carry him home as soon as he got too drunk to take care of himself. I have just seen him to-day in such a condition that he walks with difficulty and only a short distance at a time. I attribute the improvement obtained in this case, on both occasions, rather to the hygienic than to the strictly medical treatment.

